# Supplementary material for: High-pressure synthesis of seven lanthanum hydrides with a significant variability of hydrogen content
Source: Nat Commun. 2022 Nov 16;13:6987. doi: 10.1038/s41467-022-34755-y (PMC9669027; doi:10.1038/s41467-022-34755-y)
Supplement: Supplementary file 1 — Supplementary Information [file 41467_2022_34755_MOESM1_ESM.pdf]

**Supplementary Information**

# High-Pressure Synthesis of Seven Lanthanum Hydrides with a Significant Variability of Hydrogen Content

Laniel *et al*.

## Supplementary Discussion

Here after is a detailed structural description of all La-H phases. The $LaH_3$ compound was only observed after a diamond anvil from sample 1 ruptured upon attempting to increase the pressure beyond 176 GPa. The pressure abruptly dropped to about 50 GPa, and no traces of the $LaH_{9+\delta}$ and $LaH_{10+\delta}$ phases, observed at 176 GPa, could be detected. The collected SCXRD data (see Table 1 and Supplementary Table 2) shows that the structure of $LaH_3$ matches that resulting from previous structural refinements based on powder diffraction data[1]. It is interesting to note that $LaH_3$ was observed from Drozdov *et al.* after laser-heating at pressures of 152 and 178 GPa[2].

The $LaH_{\sim 4}$ solid was found in samples 1 and 3 after laser-heating at 140 and 155 GPa, respectively. From SCXRD, it was found to have an orthorhombic unit cell (*Cmcm* space group, #63) that contains four lanthanum atoms (4c Wyckoff site). Its structure is drawn in Figure 1 (b), and full crystallographic data in Supplementary Tables 3-4. The lanthanum atoms are in a four-fold coordination with interatomic distances of 2.8132(12) and 2.949(4) Å and a volume per La atom of 23.91(1) $Å^3$ at 140 GPa. $LaH_{\sim 3}$ is the only lanthanum hydride which adopts a structure type previously unobserved.

Laser-heating of paraffin and lanthanum (samples 1 and 3) at 140, 150 and 155 GPa resulted in the formation of $LaH_{4+\delta}$. It was found to have a tetragonal unit cell (*I*4/*mmm* space group, #139) containing one crystallographically distinct lanthanum atom on the 2a Wyckoff site (see Figure 1 (c) and Supplementary Tables 5-7). Each lanthanum atom has four La first neighbours at distances of 2.9417(12) Å, at 140 GPa, and a volume per lanthanum atom of 26.09(2) $Å^3$; slightly larger than for $LaH_{\sim 3}$.

The $La_4H_{23}$ solid was synthesized laser-heating at pressures 96, 106, 140 and 150 GPa (sample 1 and sample 2). Single-crystal X-ray diffraction measurements revealed its unit cell to be cubic (space group *Pm*-3*n*, #223), with eight lanthanum atoms split between the 6d and 2a Wyckoff positions, La1 and La2, respectively (see Figure 1 (d) and Supplementary Tables 8-11). The volume per lanthanum atoms is 27.986(6) Å at 150 GPa. They each have 12 neighbors, at distances of 3.3061(4), 3.3945(4) or 3.7184(3) Å at 150 GPa. The 46 hydrogen atoms per unit cell occupy the 6c, 16i and 24k Wyckoff sites. They are forming two different types of cages around the La1 and La2 atoms, with La-H distances ranging between 2.14 and 2.23 Å at 96 GPa. The La1 atoms is at the center of $H_{24}$ cages while La2 atoms are surrounded by $H_{20}$ cages. The $H_{24}$ cage is comprised of $2H_6$ as well as $12H_5$ rings and the $H_{20}$ cage is composed of $12H_5$ rings. The H-H distances in the first coordination sphere of these atoms are between 1.31 and 1.48 Å with the exception of one H atom which forms an $H_2$ dimer with an intramolecular bond length of 0.91 Å[3]. The structure type adopted by $La_4H_{23}$, *i.e.* the $Na_4Si_{23}$ structure type[4], has previously been reported for the $Eu_4H_{23}$ solid[5].

The $LaH_{6+\delta}$ solid is the only one that has been observed exclusively at one pressure: 150 GPa, in sample 1. From SCXRD measurements, it was determined to have a cubic unit cell (space group *Im*-3*m*, #229) containing two crystallographically identical lanthanum atoms on the 2a Wyckoff site, forming a *bcc* arrangement (see Figure 1 (e) and Supplementary Table 12). At 150

GPa, each lanthanum atom has eight La-La contacts of 3.3615(5) Å and with a volume per La atom of 29.24(2) $Å^3$.

Laser-heating sample 1 at 140 and 176 GPa formed the $LaH_{9+\delta}$ solid. It was experimentally determined to have a hexagonal unit cell (space group $P6_3/mmc$, #194). The unit cell is comprised of two crystallographically identical lanthanum atoms on the 2c Wyckoff site (see Figure 1 (f) and Supplementary Tables 13-14), which form a distorted *hcp* packing with six La-La distances of 3.561(2) and six others at 3.772(2) Å at 140 GPa. The volume per lanthanum atom in $LaH_{9+\delta}$ is of 35.63(2) $Å^3$ at 140 GPa. The hexagonal $LaH_{10}$ phase mentioned in ref.[2] has the same metal sublattice as $LaH_{9+\delta}$.

The $LaH_{10+\delta}$ compound was observed under the same conditions as the $LaH_{9+\delta}$ solid: it was produced in sample 1 at 140 and 176 GPa after its laser-heating. The $LaH_{10+\delta}$ compound was determined from SCXRD data to have a cubic unit cell (*Fm*-3*m* space group, #225), with a sole crystallographically unique La atom occupying the 4b Wyckoff position (see Figure 1 (g) and Supplementary Tables 15-16). At 140 GPa, the shortest La-La distance is of 3.6934(7) Å, with lanthanum atoms having twelve closest neighbours, as expected from its *fcc* arrangement. The volume per lanthanum atom is of 35.63(2) $Å^3$ at the same pressure. The demonstrated stability of $LaH_{10+\delta}$ at 140 GPa contrasts with previous measurements which detected a *C*2/*m* unit cell below 160 GPa upon the decompression of *Fm*-3*m* $LaH_{10}$ from both 172(5) and 169(10) GPa[6]. This suggests that the *Fm*-3*m* → *C*2/*m* transformation of the metal sublattice is due to non-hydrostatic stress conditions under decompression.

**Supplementary Tables**

Supplementary Table 1: Phases detected by single-crystal X-ray diffraction after sample laser-heating. The identification of a single phase in sample 2 at 106 GPa does not imply that the sample was homogeneous but rather that the other phases present could not be unambiguously identified.

| Sample | Pressure (GPa) | Maximum laser-heating temperature (K) | Phases |
|---|---|---|---|
| 1 | 96 | 2300(200) | $La_4H_{23}$, $LaCH_2$ |
| 1 | 140 | 2600(200) | $LaH_{\sim 4}$, $LaH_{4+\delta}$, $La_4H_{23}$, $LaH_{9+\delta}$, $LaH_{10+\delta}$ |
| 1 | 150 | 2500(200) | $LaH_{4+\delta}$, $La_4H_{23}$, $LaH_{6+\delta}$, LaC |
| 1 | 176 | 2500(200) | $LaH_{9+\delta}$, $LaH_{10+\delta}$ |
| | | | |
| 2 | 106 | 2800(200) | $La_4H_{23}$ |
| | | | |
| 3 | 155 | 3200(200) | $LaH_{\sim 4}$, $LaH_{4+\delta}$ |

Supplementary Table 2: Experimental crystallographic data for $LaH_3$ at 50 GPa. The corresponding cif file can be accessed from the CCDC database using the identifier CSD 2196055.

<table>
<tr><td colspan="2">Stoichiometry</td><td colspan="4">$LaH_3$</td></tr>
<tr><td colspan="2">Pressure (GPa)</td><td colspan="4">150</td></tr>
<tr><td colspan="2">Space group</td><td colspan="4">*Fm*-3*m*</td></tr>
<tr><td colspan="2">$a$ (Å)</td><td colspan="4">5.019(3)</td></tr>
<tr><td colspan="2">$b$ (Å)</td><td colspan="4">5.019(3)</td></tr>
<tr><td colspan="2">$c$ (Å)</td><td colspan="4">5.019(3)</td></tr>
<tr><td colspan="2">$V$ (Å$^3$)</td><td colspan="4">126.43(13)</td></tr>
<tr><td colspan="2">$Z$</td><td colspan="4">4</td></tr>
<tr><td colspan="2">Radiation type and wavelength</td><td colspan="4">Synchrotron, $\lambda$ = 0.29521 Å</td></tr>
<tr><td colspan="2">$\mu$ (mm$^{-1}$)</td><td colspan="4">16.81</td></tr>
<tr><td colspan="2">Number of measured/independent reflections ($I \geq 3\sigma$)</td><td colspan="4">45 / 19 (18)</td></tr>
<tr><td colspan="2">$R_{int}$</td><td colspan="4">0.1391</td></tr>
<tr><td colspan="2">$(\sin \theta/\lambda)_{max}$ (Å$^{-1}$)</td><td colspan="4">0.822</td></tr>
<tr><td colspan="2">Final R indexes ($I \geq 3\sigma$)</td><td colspan="4">$R_1$ = 0.1224; $wR_2$ = 0.1257</td></tr>
<tr><td colspan="2">Final R indexes (all data)</td><td colspan="4">$R_1$ = 0.1256; $wR_2$ = 0.1259</td></tr>
<tr><td colspan="2">GoF</td><td colspan="4">4.86</td></tr>
<tr><td colspan="2">Number of refined parameters</td><td colspan="4">2</td></tr>
<tr><td colspan="2">$\Delta\rho_{min}$, $\Delta\rho_{max}$ (eÅ$^{-3}$)</td><td colspan="4">-3.11, 2.69</td></tr>
<tr><td colspan="2"></td><td colspan="4"></td></tr>
<tr><td>**Atom name**</td><td>**Wyckoff position**</td><td colspan="3">**Fractional atomic coordinates (*x*; *y*; *z*)**</td><td>$U_{ani}$</td></tr>
<tr><td>La1</td><td>4a</td><td>0</td><td>0</td><td>0</td><td>0.0269(17)</td></tr>
</table>

Supplementary Table 3: Experimental crystallographic data for $LaH_{\sim 4}$ at 140 GPa. The corresponding cif file can be accessed from the CCDC database using the identifier CSD 2196056.

| | |
|---|---|
| Stoichiometry | $LaH_{\sim 4}$ |
| Pressure (GPa) | 140 |
| Space group | *Cmcm* |
| *a* (Å) | 2.949(4) |
| *b* (Å) | 6.7789(19) |
| *c* (Å) | 4.7837(18) |
| *V* (Å$^3$) | 95.63(14) |
| *Z* | 4 |
| Radiation type and wavelength | Synchrotron, λ = 0.29521 Å |
| μ (mm$^{-1}$) | 22.047 |
| Number of measured/independent reflections (I ≥ 3$\sigma$) | 156 / 71 (66) |
| $R_{int}$ | 0.0401 |
| $(\sin\theta/\lambda)_{max}$ (Å$^{-1}$) | 0.855 |
| Final R indexes (I ≥ 3$\sigma$) | $R_1$ = 0.0441; $wR_2$ = 0.0575 |
| Final R indexes (all data) | $R_1$ = 0.0453; $wR_2$ = 0.0576 |
| GoF | 2.86 |
| Number of refined parameters | 5 |
| $\Delta\rho_{min}$, $\Delta\rho_{max}$ (eÅ$^{-3}$) | -2.32, 4.23 |
| | |

| **Atom name** | **Wyckoff position** | **Fractional atomic coordinates (*x*; *y*; *z*)** | | | $U_{ani}$ |
|---|---|---|---|---|---|
| La1 | 4c | 1/2 | 0.60926(19) | 3/4 | 0.0123(9) |

Supplementary Table 4: Experimental crystallographic data for $LaH_{\sim4}$ at 155 GPa. The corresponding cif file can be accessed from the CCDC database using the identifier CSD 2196057.

| | | | | | |
|---|---|---|---|---|---|
| Stoichiometry | | $LaH_{\sim4}$ | | | |
| Pressure (GPa) | | 155 | | | |
| Space group | | *Cmcm* | | | |
| $a$ (Å) | | 2.850(4) | | | |
| $b$ (Å) | | 6.7340(19) | | | |
| $c$ (Å) | | 4.5504(18) | | | |
| $V$ (Å$^3$) | | 87.32(13) | | | |
| $Z$ | | 4 | | | |
| Radiation type and wavelength | | Synchrotron, $\lambda$ = 0.29521 Å | | | |
| $\mu$ (mm$^{-1}$) | | 24.146 | | | |
| Number of measured/independent reflections ($I \geq 3\sigma$) | | 101 / 58 (44) | | | |
| $R_{int}$ | | 0.0534 | | | |
| $(\sin\theta/\lambda)_{max}$ (Å$^{-1}$) | | 0.794 | | | |
| Final R indexes ($I \geq 3\sigma$) | | $R_1$ = 0.0503; $wR_2$ = 0.506 | | | |
| Final R indexes (all data) | | $R_1$ = 0.706; $wR_2$ = 0.0510 | | | |
| GoF | | 2.46 | | | |
| Number of refined parameters | | 5 | | | |
| $\Delta\rho_{min}$, $\Delta\rho_{max}$ (eÅ$^{-3}$) | | -3.02, 3.63 | | | |
| | | | | | |
| **Atom name** | **Wyckoff position** | **Fractional atomic coordinates (*x*; *y*; *z*)** | | | $U_{ani}$ |
| La1 | 4c | 1/2 | 0.6093(5) | 3/4 | 0.0114(8) |

Supplementary Table 5: Experimental crystallographic data for $LaH_{4+\delta}$ at 140 GPa. The corresponding cif file can be accessed from the CCDC database using the identifier CSD 2196059.

| | | | | | |
|---|---|---|---|---|---|
| Stoichiometry | | $LaH_{4+\delta}$ | | | |
| Pressure (GPa) | | 140 | | | |
| Space group | | *I4/mmm* | | | |
| $a$ (Å) | | 2.9418(12) | | | |
| $b$ (Å) | | 2.9418(12) | | | |
| $c$ (Å) | | 6.028(3) | | | |
| $V$ (Å$^3$) | | 52.17(4) | | | |
| $Z$ | | 2 | | | |
| Radiation type and wavelength | | Synchrotron, λ = 0.29521 Å | | | |
| μ (mm$^{-1}$) | | 20.046 | | | |
| Number of measured/independent reflections (I ≥ 3$\sigma$) | | 84 / 42 (42) | | | |
| $R_{int}$ | | 0.0166 | | | |
| $(\sin\theta/\lambda)_{max}$ (Å$^{-1}$) | | 0.864 | | | |
| Final R indexes (I ≥ 3$\sigma$) | | $R_1$ = 0.0400; $wR_2$ = 0.0478 | | | |
| Final R indexes (all data) | | $R_1$ = 0.0400; $wR_2$ = 0.0478 | | | |
| GoF | | 2.44 | | | |
| Number of refined parameters | | 3 | | | |
| $\Delta\rho_{min}$, $\Delta\rho_{max}$ (eÅ$^{-3}$) | | -3.31, 3.01 | | | |
| | | | | | |
| **Atom name** | **Wyckoff position** | **Fractional atomic coordinates (*x*; *y*; *z*)** | | | $U_{ani}$ |
| La1 | 2a | 1/2 | 1/2 | 1/2 | 0.0106(4) |

Supplementary Table 6: Experimental crystallographic data for $LaH_{4+\delta}$ at 150 GPa. The corresponding cif file can be accessed from the CCDC database using the identifier CSD 2196058.

| Stoichiometry | | $LaH_{4+\delta}$ | | | |
|---|---|---|---|---|---|
| Pressure (GPa) | | 150 | | | |
| Space group | | *I4/mmm* | | | |
| *a* (Å) | | 2.882(2) | | | |
| *b* (Å) | | 2.882(2) | | | |
| *c* (Å) | | 6.031(5) | | | |
| *V* (Å$^3$) | | 50.11(6) | | | |
| *Z* | | 2 | | | |
| Radiation type and wavelength | | Synchrotron, λ = 0.29521 Å | | | |
| μ (mm$^{-1}$) | | 20.868 | | | |
| Number of measured/independent reflections (I ≥ 3σ) | | 50 / 26 (26) | | | |
| $R_{int}$ | | 0.1059 | | | |
| $(\sin\theta/\lambda)_{max}$ (Å$^{-1}$) | | 0.861 | | | |
| Final R indexes (I ≥ 3σ) | | $R_1$ = 0.0613; $wR_2$ = 0.0729 | | | |
| Final R indexes (all data) | | $R_1$ = 0.0613; $wR_2$ = 0.0729 | | | |
| GoF | | 3.15 | | | |
| Number of refined parameters | | 3 | | | |
| $\Delta\rho_{min}$, $\Delta\rho_{max}$ (eÅ$^{-3}$) | | -3.2, 2.7 | | | |
| | | | | | |
| **Atom name** | **Wyckoff position** | **Fractional atomic coordinates (*x*; *y*; *z*)** | | | $U_{ani}$ |
| La1 | 2a | 1/2 | 1/2 | 1/2 | 0.0104(10) |

Supplementary Table 7: Experimental crystallographic data for $LaH_{4+\delta}$ at 155 GPa. The corresponding cif file can be accessed from the CCDC database using the identifier CSD 2196060.

| | | | | | |
|---|---|---|---|---|---|
| Stoichiometry | | $LaH_{4+\delta}$ | | | |
| Pressure (GPa) | | 155 | | | |
| Space group | | *I4/mmm* | | | |
| *a* (Å) | | 2.8391(5) | | | |
| *b* (Å) | | 2.8391(5) | | | |
| *c* (Å) | | 6.0093(10) | | | |
| *V* (Å$^3$) | | 48.438(15) | | | |
| *Z* | | 2 | | | |
| Radiation type and wavelength | | Synchrotron, λ = 0.29521 Å | | | |
| μ (mm$^{-1}$) | | 21.59 | | | |
| Number of measured/independent reflections (I ≥ 3$\sigma$) | | 61 / 26 (26) | | | |
| $R_{int}$ | | 0.0195 | | | |
| $(\sin\theta/\lambda)_{max}$ (Å$^{-1}$) | | 0.786 | | | |
| Final R indexes (I ≥ 3$\sigma$) | | $R_1$ = 0.0380; $wR_2$ = 0.0470 | | | |
| Final R indexes (all data) | | $R_1$ = 0.0380; $wR_2$ = 0.0470 | | | |
| GoF | | 3.37 | | | |
| Number of refined parameters | | 3 | | | |
| $\Delta\rho_{min}$, $\Delta\rho_{max}$ (eÅ$^{-3}$) | | -2.0, 2.0 | | | |
| | | | | | |
| **Atom name** | **Wyckoff position** | **Fractional atomic coordinates (*x*; *y*; *z*)** | | | $U_{ani}$ |
| La1 | 2a | 1/2 | 1/2 | 1/2 | 0.0150(6) |

Supplementary Table 8: Experimental crystallographic data for $La_4H_{23}$ at 96 GPa. The corresponding cif file can be accessed from the CCDC database using the identifier CSD 2196061. The position of the H atoms has been determined from a preliminary experimental model then relaxed with DFT calculations. The relaxed values are those provided here.

| | Exp. | Calc. |
|---|---|---|
| Stoichiometry | $La_4H_{23}$ | $La_4H_{23}$ |
| Pressure (GPa) | 96 | 96 |
| Space group | *Pm*-3*n* | *Pm*-3*n* |
| *a* (Å) | 6.170(3) | 6.1685 |
| *b* (Å) | 6.170(3) | 6.1685 |
| *c* (Å) | 6.170(3) | 6.1685 |
| *V* (Å$^3$) | 234.86(16) | 234.714 |
| *Z* | 8 | 8 |
| Radiation type and wavelength | Synchrotron, λ = 0.29521 Å | |
| μ (mm$^{-1}$) | 17.954 | |
| Number of measured/independent reflections (I ≥ 3$\sigma$) | 687 / 112 (55) | |
| $R_{int}$ | 0.0537 | |
| (sin θ/λ)$_{max}$ (Å$^{-1}$) | 0.869 | |
| Final R indexes (I ≥ 3$\sigma$) | $R_1$ = 0.0209; $wR_2$ = 0.0248 | |
| Final R indexes (all data) | $R_1$ = 0.0455; $wR_2$ = 0.0278 | |
| GoF | 1.57 | |
| Number of refined parameters | 4 | |
| $\Delta\rho_{min}$, $\Delta\rho_{max}$ (eÅ$^{-3}$) | -1.62, 1.85 | |
| | | |

| **Atom name** | **Wyckoff position** | **Fractional atomic coordinates (*x*; *y*; *z*)** | | | $U_{ani}$ |
|---|---|---|---|---|---|
| La1 | 6c | 1/2 | 1/4 | 0 | 0.00502(19) |
| La2 | 2a | 0 | 0 | 0 | 0.0069(2) |
| H1 | 6c | 1/4 | 0 | 1/2 | -- |
| H2 | 16i | 0.2074 | 0.2074 | 0.2074 | -- |
| H3 | 24k | 0 | 0.3126 | 0.1506 | -- |

Supplementary Table 9: Experimental crystallographic data for $La_4H_{23}$ at 106 GPa. The corresponding cif file can be accessed from the CCDC database using the identifier CSD 2196062. The position of the H atoms was not determined (experimentally or theoretically) at this pressure.

| Stoichiometry | | $La_4H_{23}$ | | | |
|---|---|---|---|---|---|
| Pressure (GPa) | | 106 | | | |
| Space group | | *Pm*-3*n* | | | |
| *a* (Å) | | 6.1152(8) | | | |
| *b* (Å) | | 6.1152(8) | | | |
| *c* (Å) | | 6.1152(8) | | | |
| *V* (Å$^3$) | | 228.68(5) | | | |
| *Z* | | 8 | | | |
| Radiation type and wavelength | | Synchrotron, λ = 0.29521 Å | | | |
| μ (mm$^{-1}$) | | 17.614 | | | |
| Number of measured/independent reflections ($I \geq 3\sigma$) | | 679 / 100 (51) | | | |
| $R_{int}$ | | 0.1091 | | | |
| $(\sin \theta/\lambda)_{max}$ (Å$^{-1}$) | | 0.765 | | | |
| Final R indexes ($I \geq 3\sigma$) | | $R_1$ = 0.0339; $wR_2$ = 0.0418 | | | |
| Final R indexes (all data) | | $R_1$ = 0.0511; $wR_2$ = 0.0431 | | | |
| GoF | | 1.42 | | | |
| Number of refined parameters | | 4 | | | |
| $\Delta\rho_{min}$, $\Delta\rho_{max}$ (eÅ$^{-3}$) | | -1.98, 3.47 | | | |
| | | | | | |
| **Atom name** | **Wyckoff position** | **Fractional atomic coordinates (*x*; *y*; *z*)** | | | $U_{ani}$ |
| La1 | 6c | 1/2 | 1/4 | 0 | 0.0084(4) |
| La2 | 2a | 0 | 0 | 0 | 0.0129(4) |

Supplementary Table 10: Experimental crystallographic data for $La_4H_{23}$ at 140 GPa. The corresponding cif file can be accessed from the CCDC database using the identifier CSD 2196064. The position of the H atoms was not determined (experimentally or theoretically) at this pressure.

| | | | | | |
|---|---|---|---|---|---|
| Stoichiometry | | $La_4H_{23}$ | | | |
| Pressure (GPa) | | 140 | | | |
| Space group | | *Pm*-3*n* | | | |
| *a* (Å) | | 5.952(3) | | | |
| *b* (Å) | | 5.952(3) | | | |
| *c* (Å) | | 5.952(3) | | | |
| *V* ($Å^3$) | | 210.86(18) | | | |
| *Z* | | 8 | | | |
| Radiation type and wavelength | | Synchrotron, λ = 0.29521 Å | | | |
| μ ($mm^{-1}$) | | 20.038 | | | |
| Number of measured/independent reflections (I ≥ 3$\sigma$) | | 284 / 54 (32) | | | |
| $R_{int}$ | | 0.1076 | | | |
| $(\sin \theta/\lambda)_{max}$ ($Å^{-1}$) | | 0.661 | | | |
| Final R indexes (I ≥ 3$\sigma$) | | $R_1$ = 0.0595; $wR_2$ = 0.0522 | | | |
| Final R indexes (all data) | | $R_1$ = 0.0903; $wR_2$ = 0.0556 | | | |
| GoF | | 1.52 | | | |
| Number of refined parameters | | 4 | | | |
| $\Delta\rho_{min}$, $\Delta\rho_{max}$ ($eÅ^{-3}$) | | -2.29, 3.89 | | | |
| | | | | | |
| **Atom name** | **Wyckoff position** | **Fractional atomic coordinates (*x*; *y*; *z*)** | | | ***$U_{ani}$*** |
| La1 | 6c | 1/2 | 1/4 | 0 | 0.0199(10) |
| La2 | 2a | 0 | 0 | 0 | 0.0357(11) |

Supplementary Table 11: Experimental crystallographic data for $La_4H_{23}$ at 150 GPa. The corresponding cif file can be accessed from the CCDC database using the identifier CSD 2196063. The position of the H atoms was not determined (experimentally or theoretically) at this pressure.

| | | | | | |
|---|---|---|---|---|---|
| Stoichiometry | | $La_4H_{23}$ | | | |
| Pressure (GPa) | | 150 | | | |
| Space group | | *Pm*-3*n* | | | |
| *a* (Å) | | 6.0722(8) | | | |
| *b* (Å) | | 6.0722(8) | | | |
| *c* (Å) | | 6.0722(8) | | | |
| $V$ (Å$^3$) | | 223.89(5) | | | |
| *Z* | | 8 | | | |
| Radiation type and wavelength | | Synchrotron, $\lambda = 0.29521$ Å | | | |
| $\mu$ (mm$^{-1}$) | | 17.614 | | | |
| Number of measured/independent reflections ($I \geq 3\sigma$) | | 489 / 113 (90) | | | |
| $R_{int}$ | | 0.0486 | | | |
| $(\sin \theta/\lambda)_{max}$ (Å$^{-1}$) | | 0.890 | | | |
| Final R indexes ($I \geq 3\sigma$) | | $R_1 = 0.0329$; $wR_2 = 0.0384$ | | | |
| Final R indexes (all data) | | $R_1 = 0.0416$; $wR_2 = 0.0390$ | | | |
| GoF | | 2.37 | | | |
| Number of refined parameters | | 4 | | | |
| $\Delta\rho_{min}$, $\Delta\rho_{max}$ (eÅ$^{-3}$) | | -2.21, 2.42 | | | |
| | | | | | |
| **Atom name** | **Wyckoff position** | **Fractional atomic coordinates (*x*; *y*; *z*)** | | | $U_{ani}$ |
| La1 | 6c | 1/2 | 1/4 | 0 | 0.0091(2) |
| La2 | 2a | 0 | 0 | 0 | 0.0309(3) |

Supplementary Table 12: Experimental crystallographic data for $LaH_{6+\delta}$ at 150 GPa. The corresponding cif file can be accessed from the CCDC database using the identifier CSD 2196065.

| | | | | | |
|---|---|---|---|---|---|
| Stoichiometry | | $LaH_{6+\delta}$ | | | |
| Pressure (GPa) | | 150 | | | |
| Space group | | *Im*-3*m* | | | |
| *a* (Å) | | 3.8710(10) | | | |
| *b* (Å) | | 3.8710(10) | | | |
| *c* (Å) | | 3.8710(10) | | | |
| *V* ($Å^3$) | | 58.01(3) | | | |
| *Z* | | 2 | | | |
| Radiation type and wavelength | | Synchrotron, λ = 0.29521 Å | | | |
| μ ($mm^{-1}$) | | 18.32 | | | |
| Number of measured/independent reflections (I ≥ 3$\sigma$) | | 50 / 18 (18) | | | |
| $R_{int}$ | | 0.0674 | | | |
| $(\sin \theta/\lambda)_{max}$ ($Å^{-1}$) | | 0.796 | | | |
| Final R indexes (I ≥ 3$\sigma$) | | $R_1$ = 0.0490; $wR_2$ = 0.0620 | | | |
| Final R indexes (all data) | | $R_1$ = 0.0490; $wR_2$ = 0.0620 | | | |
| GoF | | 3.18 | | | |
| Number of refined parameters | | 2 | | | |
| $\Delta\rho_{min}$, $\Delta\rho_{max}$ ($eÅ^{-3}$) | | -1.95, 3.88 | | | |
| | | | | | |
| **Atom name** | **Wyckoff position** | **Fractional atomic coordinates (*x*; *y*; *z*)** | | | $U_{ani}$ |
| La1 | 2a | 0 | 0 | 0 | 0.0130(8) |

Supplementary Table 13: Experimental crystallographic data for $LaH_{9+\delta}$ at 140 GPa. The corresponding cif file can be accessed from the CCDC database using the identifier CSD 2196066.

| | | | | | |
|---|---|---|---|---|---|
| Stoichiometry | | $LaH_{9+\delta}$ | | | |
| Pressure (GPa) | | 140 | | | |
| Space group | | $P6_3/mmc$ | | | |
| $a$ (Å) | | 3.772(2) | | | |
| $b$ (Å) | | 3.772(2) | | | |
| $c$ (Å) | | 5.634(4) | | | |
| $\beta$ (°) | | 120 | | | |
| $V$ (Å$^3$) | | 69.41(7) | | | |
| $Z$ | | 2 | | | |
| Radiation type and wavelength | | Synchrotron, $\lambda$ = 0.29521 Å | | | |
| $\mu$ (mm$^{-1}$) | | 15.128 | | | |
| Number of measured/independent reflections ($I \geq 3\sigma$) | | 179 / 62 (58) | | | |
| $R_{int}$ | | 0.023 | | | |
| $(\sin \theta/\lambda)_{max}$ (Å$^{-1}$) | | 0.881 | | | |
| Final R indexes ($I \geq 3\sigma$) | | $R_1$ = 0.0353; $wR_2$ = 0.0415 | | | |
| Final R indexes (all data) | | $R_1$ = 0.0356; $wR_2$ = 0.0415 | | | |
| GoF | | 2.30 | | | |
| Number of refined parameters | | 3 | | | |
| $\Delta\rho_{min}$, $\Delta\rho_{max}$ (eÅ$^{-3}$) | | -2.46, 2.18 | | | |
| | | | | | |
| **Atom name** | **Wyckoff position** | **Fractional atomic coordinates (*x*; *y*; *z*)** | | | ***U**ani* |
| La1 | 2d | 2/3 | 1/3 | 3/4 | 0.0099(4) |

Supplementary Table 14: Experimental crystallographic data for $LaH_{9+\delta}$ at 176 GPa. The corresponding cif file can be accessed from the CCDC database using the identifier CSD 2196067.

<table>
<tr><td colspan="2">Stoichiometry</td><td colspan="4">$LaH_{9+\delta}$</td></tr>
<tr><td colspan="2">Pressure (GPa)</td><td colspan="4">176</td></tr>
<tr><td colspan="2">Space group</td><td colspan="4">$P6_3/mmc$</td></tr>
<tr><td colspan="2">$a$ (Å)</td><td colspan="4">3.7589(10)</td></tr>
<tr><td colspan="2">$b$ (Å)</td><td colspan="4">3.7589(10)</td></tr>
<tr><td colspan="2">$c$ (Å)</td><td colspan="4">5.583(2)</td></tr>
<tr><td colspan="2">$\beta$ (°)</td><td colspan="4">120</td></tr>
<tr><td colspan="2">$V$ (Å$^3$)</td><td colspan="4">68.31(4)</td></tr>
<tr><td colspan="2">$Z$</td><td colspan="4">2</td></tr>
<tr><td colspan="2">Radiation type and wavelength</td><td colspan="4">Synchrotron, $\lambda$ = 0.29521 Å</td></tr>
<tr><td colspan="2">$\mu$ (mm$^{-1}$)</td><td colspan="4">15.37</td></tr>
<tr><td colspan="2">Number of measured/independent reflections ($I \geq 3\sigma$)</td><td colspan="4">196 / 69 (62)</td></tr>
<tr><td colspan="2">$R_{int}$</td><td colspan="4">0.0242</td></tr>
<tr><td colspan="2">$(\sin \theta/\lambda)_{max}$ (Å$^{-1}$)</td><td colspan="4">0.874</td></tr>
<tr><td colspan="2">Final R indexes ($I \geq 3\sigma$)</td><td colspan="4">$R_1$ = 0.0243; $wR_2$ = 0.0284</td></tr>
<tr><td colspan="2">Final R indexes (all data)</td><td colspan="4">$R_1$ = 0.0326; $wR_2$ = 0.0285</td></tr>
<tr><td colspan="2">GoF</td><td colspan="4">1.76</td></tr>
<tr><td colspan="2">Number of refined parameters</td><td colspan="4">3</td></tr>
<tr><td colspan="2">$\Delta\rho_{min}$, $\Delta\rho_{max}$ (eÅ$^{-3}$)</td><td colspan="4">-1.68, 1.37</td></tr>
<tr><td colspan="2"></td><td colspan="4"></td></tr>
<tr><td>Atom name</td><td>Wyckoff position</td><td colspan="3">Fractional atomic coordinates ($x$; $y$; $z$)</td><td>$U_{ani}$</td></tr>
<tr><td>La1</td><td>2d</td><td>2/3</td><td>1/3</td><td>3/4</td><td>0.0104(3)</td></tr>
</table>

Supplementary Table 15: Experimental crystallographic data for $LaH_{10+\delta}$ at 140 GPa. The corresponding cif file can be accessed from the CCDC database using the identifier CSD 2196069.

| | | | | | |
|---|---|---|---|---|---|
| Stoichiometry | | $LaH_{10+\delta}$ | | | |
| Pressure (GPa) | | 140 | | | |
| Space group | | *Fm*-3*m* | | | |
| $a$ (Å) | | 5.2233(14) | | | |
| $b$ (Å) | | 5.2233(14) | | | |
| $c$ (Å) | | 5.2233(14) | | | |
| $V$ (Å$^3$) | | 142.51(7) | | | |
| $Z$ | | 4 | | | |
| Radiation type and wavelength | | Synchrotron, λ = 0.29521 Å | | | |
| μ (mm$^{-1}$) | | 14.913 | | | |
| Number of measured/independent reflections ($I \geq 3\sigma$) | | 106 / 28 (27) | | | |
| $R_{int}$ | | 0.0454 | | | |
| $(\sin \theta/\lambda)_{max}$ (Å$^{-1}$) | | 0.872 | | | |
| Final R indexes ($I \geq 3\sigma$) | | $R_1$ = 0.0495; $wR_2$ = 0.0612 | | | |
| Final R indexes (all data) | | $R_1$ = 0.0498; $wR_2$ = 0.0612 | | | |
| GoF | | 2.65 | | | |
| Number of refined parameters | | 2 | | | |
| $\Delta\rho_{min}$, $\Delta\rho_{max}$ (eÅ$^{-3}$) | | -1.80, 4.44 | | | |
| | | | | | |
| **Atom name** | **Wyckoff position** | **Fractional atomic coordinates (*x*; *y*; *z*)** | | | $U_{ani}$ |
| La1 | 4a | 0 | 0 | 0 | 0.0124(5) |

Supplementary Table 16: Experimental crystallographic data for $LaH_{10+\delta}$ at 176 GPa. The corresponding cif file can be accessed from the CCDC database using the identifier CSD 2196068.

| | | | | | |
|---|---|---|---|---|---|
| Stoichiometry | | $LaH_{10+\delta}$ | | | |
| Pressure (GPa) | | 176 | | | |
| Space group | | *Fm*-3*m* | | | |
| $a$ (Å) | | 5.1798(7) | | | |
| $b$ (Å) | | 5.1798(7) | | | |
| $c$ (Å) | | 5.1798(7) | | | |
| $V$ (Å$^3$) | | 138.98(3) | | | |
| $Z$ | | 4 | | | |
| Radiation type and wavelength | | Synchrotron, λ = 0.29521 Å | | | |
| μ (mm$^{-1}$) | | 15.292 | | | |
| Number of measured/independent reflections ($I \geq 3\sigma$) | | 97 / 69 (62) | | | |
| $R_{int}$ | | 0.0575 | | | |
| $(\sin \theta/\lambda)_{max}$ (Å$^{-1}$) | | 0.864 | | | |
| Final R indexes ($I \geq 3\sigma$) | | $R_1$ = 0.0428; $wR_2$ = 0.0500 | | | |
| Final R indexes (all data) | | $R_1$ = 0.0428; $wR_2$ = 0.0500 | | | |
| GoF | | 2.18 | | | |
| Number of refined parameters | | 2 | | | |
| $\Delta\rho_{min}$, $\Delta\rho_{max}$ (eÅ$^{-3}$) | | -4.65, 2.97 | | | |
| | | | | | |
| **Atom name** | **Wyckoff position** | **Fractional atomic coordinates (*x*; *y*; *z*)** | | | $U_{ani}$ |
| La1 | 4a | 0 | 0 | 0 | 0.0090(5) |

Supplementary Table 17: Experimental crystallographic data for $LaCH_2$ at 96 GPa. The position of the H atoms has been determined from a preliminary experimental model then relaxed with DFT calculations. The relaxed values are those provided here for the H atom. The corresponding cif file can be accessed from the CCDC database using the identifier CSD 2196054.

| | Experiments | DFT |
|---|---|---|
| Stoichiometry | $LaCH_2$ | $LaCH_2$ |
| Pressure (GPa) | 96 | 96 |
| Space group | $P6_3/mmc$ | $P6_3/mmc$ |
| $a$ (Å) | 3.4936(11) | 3.5607 |
| $b$ (Å) | 3.4936(11) | 3.5607 |
| $c$ (Å) | 4.943(3) | 5.097 |
| $V$ (Å$^3$) | 52.24(4) | 55.96 |
| $Z$ | 2 | 2 |
| Radiation type and wavelength | Synchrotron, $\lambda$ = 0.29521 Å | |
| $\mu$ (mm$^{-1}$) | 20.198 | |
| Number of measured/independent reflections ($I \geq 3\sigma$) | 144 / 43 (37) | |
| $R_{int}$ | 0.0287 | |
| $(\sin \theta/\lambda)_{max}$ (Å$^{-1}$) | 0.880 | |
| Final R indexes ($I \geq 3\sigma$) | $R_1$ = 0.0429; $wR_2$ = 0.0459 | |
| Final R indexes (all data) | $R_1$ = 0.0500; $wR_2$ = 0.0463 | |
| GoF | 3.31 | |
| Number of refined parameters | 5 | |
| $\Delta\rho_{min}$, $\Delta\rho_{max}$ (eÅ$^{-3}$) | -1.3, 3.7 | |
| | | |

| **Atom name** | **Wyckoff position** | **Fractional atomic coordinates (*x*; *y*; *z*)** | | | $U_{ani}$ |
|---|---|---|---|---|---|
| La1 | 2c | 1/3 | 2/3 | 1/4 | 0.0254(11) |
| C1 | 2a | 0 | 0 | 0 | 0.006(3) |
| H1 | 4f | 1/3 | 2/3 | 0.1663 | -- |

Supplementary Table 18: Experimental crystallographic data for LaC at 150 GPa. The corresponding cif file can be accessed from the CCDC database using the identifier CSD 2196053.

| Stoichiometry | | LaC | | | |
|---|---|---|---|---|---|
| Pressure (GPa) | | 150 | | | |
| Space group | | *Pm*-3*m* | | | |
| *a* (Å) | | 2.7526(8) | | | |
| *b* (Å) | | 2.7526(8) | | | |
| *c* (Å) | | 2.7526(8) | | | |
| *V* (Å$^3$) | | 20.856(10) | | | |
| *Z* | | 1 | | | |
| Radiation type and wavelength | | Synchrotron, λ = 0.29521 Å | | | |
| μ (mm$^{-1}$) | | 25.293 | | | |
| Number of measured/independent reflections (I ≥ 3$\sigma$) | | 51 / 17 (17) | | | |
| $R_{int}$ | | 0.0665 | | | |
| (sin θ/λ)$_{max}$ (Å$^{-1}$) | | 0.832 | | | |
| Final R indexes (I ≥ 3$\sigma$) | | $R_1$ = 0.0502; $wR_2$ = 0.0486 | | | |
| Final R indexes (all data) | | $R_1$ = 0.0502; $wR_2$ = 0.0486 | | | |
| GoF | | 2.61 | | | |
| Number of refined parameters | | 3 | | | |
| $\Delta\rho_{min}$, $\Delta\rho_{max}$ (eÅ$^{-3}$) | | -3.1, 2.9 | | | |
| | | | | | |
| **Atom name** | **Wyckoff position** | **Fractional atomic coordinates (*x*; *y*; *z*)** | | | $U_{ani}$ |
| La1 | 1b | 1/2 | 1/2 | 1/2 | 0.012 |
| C1 | 1a | 0 | 0 | 0 | 0.013 |

Supplementary Table 19: Second order Birch-Murnaghan equation of state parameters used for drawing the coloured bands in Figure 1, with the exception of $LaH_6$. Because of the very low number of points available, for each phase the $V_X$ was fixed to the experimental volume at the pressure X, where X is the lowest pressure that a given compound was experimentally observed. These values were solely used to draw curves to guide the eyes in Figure 1; they are of limited physical meaning due to the large uncertainty on the $K_X$ parameter.

| **Compounds** | **X (GPa)** | **$V_X$ (Å$^3$, fixed)** | **$K_X$ (GPa)** |
|---|---|---|---|
| $LaH_3$ | 50 | 126.6 | 109(3) |
| $LaH_{\sim 3}$ | 140 | 95.4 | 258(17) |
| $LaH_4$ | 140 | 52.2 | 185(16) |
| $LaH_4H_{23}$ | 96 | 234.9 | 462(104) |
| $LaH_9$ | 140 | 69.7 | 1742(48) |
| $LaH_{10}$ | 140 | 142.4 | 1415(39) |

Supplementary Table 20: Pressure in $LaH_4$ when using different xc-functionals.

| **xc-functional** | **Pressure (GPa)** |
|---|---|
| LDA | 98.1 |
| PBE | 107.9 |
| PBEsol | 100.5 |
| Am05 | 98.4 |

Supplementary Table 21: Supercell sizes and k-points used for the finite temperature calculations (Supplementary Figure 5).

| **Stoichiometry** | **Supercell size** (Number of atoms) | **K-point grid** |
|---|---|---|
| $LaH_4$ | 180 | 4x4x4 |
| $LaH_6$ | 112 | 6x6x6 |
| $LaH_9$ | 80 | 6x6x6 |
| $LaH_{10}$ | 44 | 4x4x4 |

## Supplementary Figures

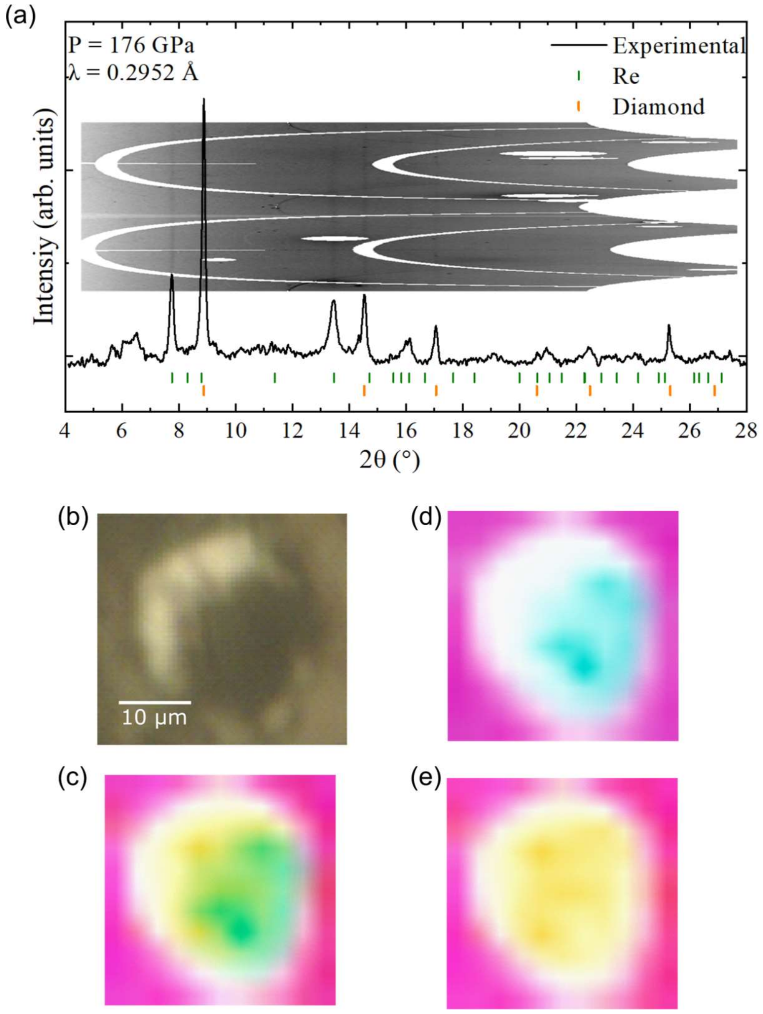


Supplementary Figure 1: X-ray diffraction data collected on sample 1 at 176 GPa. (a) Integrated X-ray diffraction pattern with diffraction lines explained by the rhenium gasket and diamond powder formed from decomposed paraffin. (Background) Corresponding X-ray diffraction pattern, where the homogeneous diffraction lines of diamond demonstrate that it is a powder. (b) Microphotograph of the same sample. (c) Image produced from the X-ray diffraction mapping of the same sample. The intensity of diffraction lines attributed to the rhenium gasket, the diamond powder and a mixture of $LaH_{9+\delta}$+$LaH_{10+\delta}$, respectively, were integrated and attributed individual colors of pink, blue and yellow. The greenish color results from the combination of blue and yellow, i.e. position where both diamond powder and $LaH_{9+\delta}$+$LaH_{10+\delta}$ are visible. (d) The same image as in (c) but with the contribution of the diamond powder removed. (e) The same image as in (c) but this time with the contribution of $LaH_{9+\delta}$+$LaH_{10+\delta}$ removed. Diamond powder is observed almost everywhere in the sample chamber where paraffin was before laser-heating—including on top of the La piece. This provides definitive evidence that it is a decomposition product of laser-heated paraffin, along with the released hydrogen.

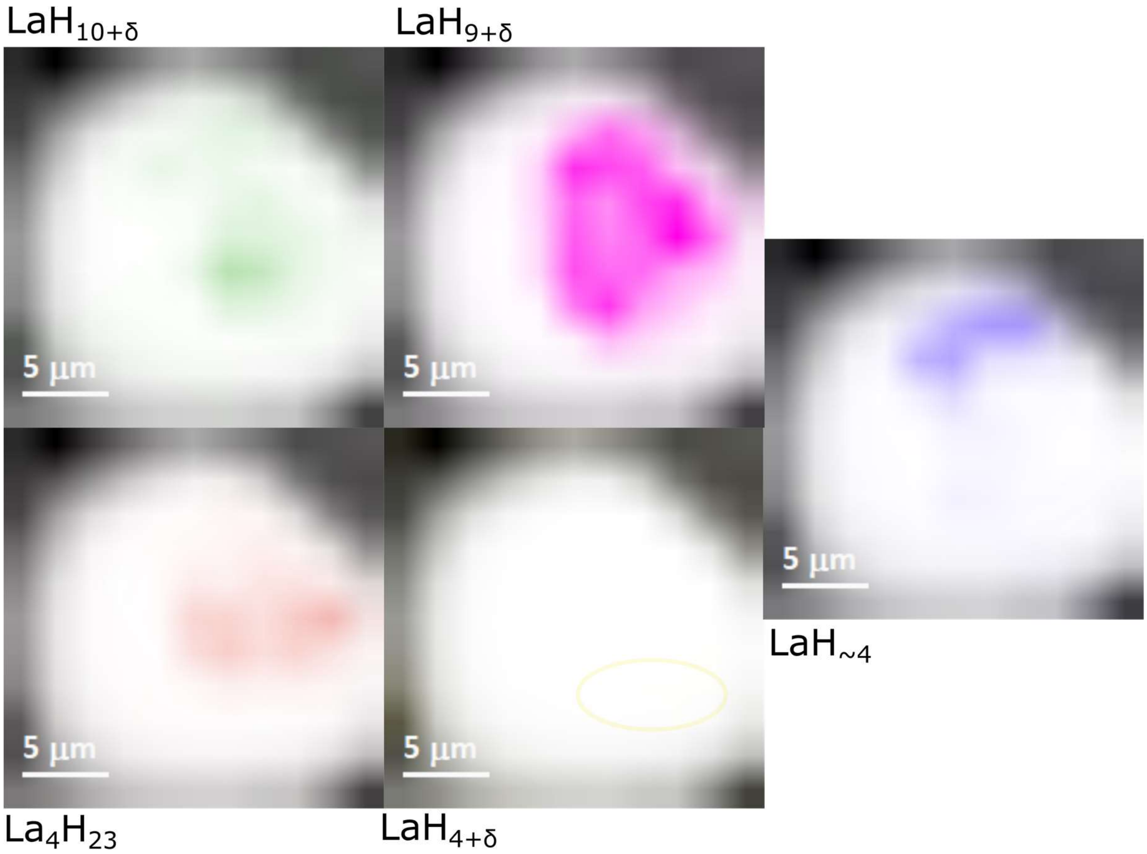


Supplementary Figure 2: Sample X-ray diffraction map at 140 GPa, collected as 11x11 images with 2 µm steps. For each phase, an X-ray diffraction line not significantly overlapping with that of another phase was selected. For $LaH_{10+\delta}$, $LaH_{9+\delta}$, $La_4H_{23}$, $LaH_{4+\delta}$ and $LaH_{\sim 4}$, the 6.48, 6.00, 5.69, 6.26 and 8.14° 2θ peaks (λ= 0.29521 Å) were selected, respectively. Each panel shows the XRD map with one phase visible, with the color's intensity being proportional to the intensity of its X-ray diffraction line. To allow comparison between images, all intensities were normalized to the most intense peak out of any phases (i.e. $LaH_{9+\delta}$). For $LaH_{4+\delta}$, the intensity of the chosen diffraction line is so weak that it is barely visible. The sample region featuring some $LaH_{4+\delta}$ is encircled in yellow. Due to the compounds' preferred orientation, these maps can solely be qualitatively interpreted.

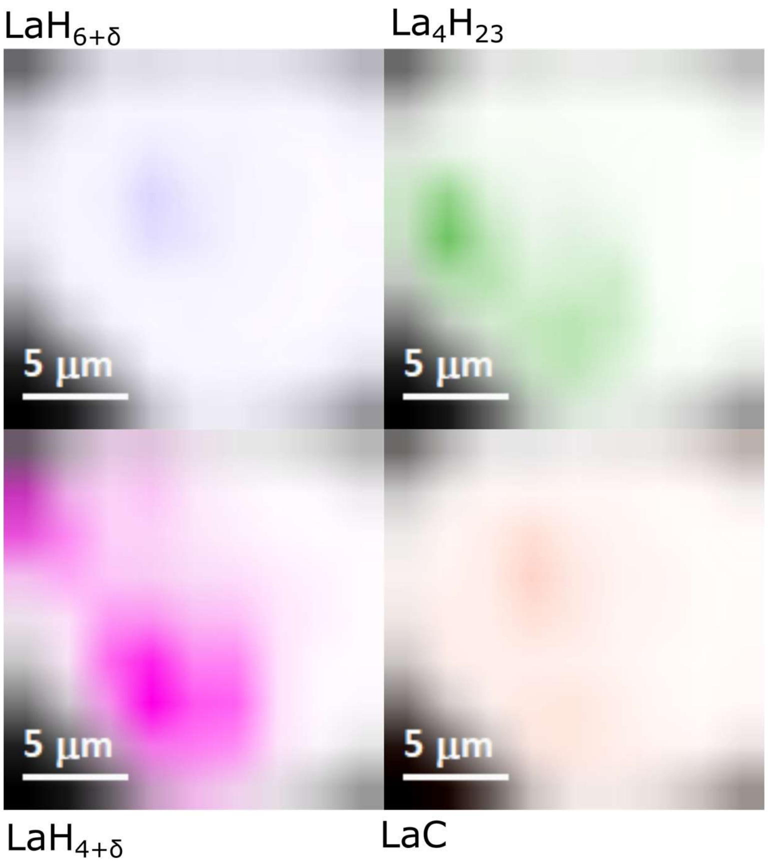


Supplementary Figure 3: Sample X-ray diffraction map at 150 GPa, collected as 9x9 images with 2 µm steps. For each phase, an X-ray diffraction line not significantly overlapping with that of another phase was selected. For $LaH_{6+\delta}$, $La_4H_{23}$, $LaH_{4+\delta}$ and LaC, the 16.40, 6.82, 6.51, and 13.77° 2θ peaks (λ= 0.29521 Å) were selected, respectively. Each panel shows the XRD map with one phase visible, with the color's intensity being proportional to the intensity of its X-ray diffraction line. To allow comparison between images, all intensities were normalized to the most intense peak out of any phases (*i.e.* $LaH_{4+\delta}$). Due to preferred orientation, selected peaks chosen at high and low 2θ values, these maps can solely be used for a qualitative interpretation.

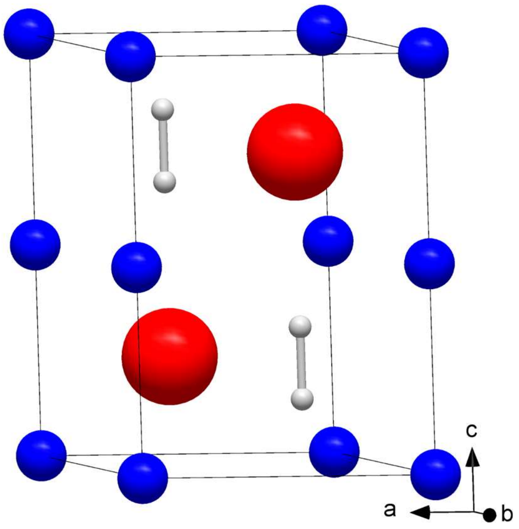


Supplementary Figure 4: Crystal structure of $LaCH_2$ at 96 GPa. The red, blue and white spheres represent lanthanum, carbon and hydrogen atoms, respectively. The position of hydrogen atoms was determined from DFT calculations, while that of La and C from the experimental data.

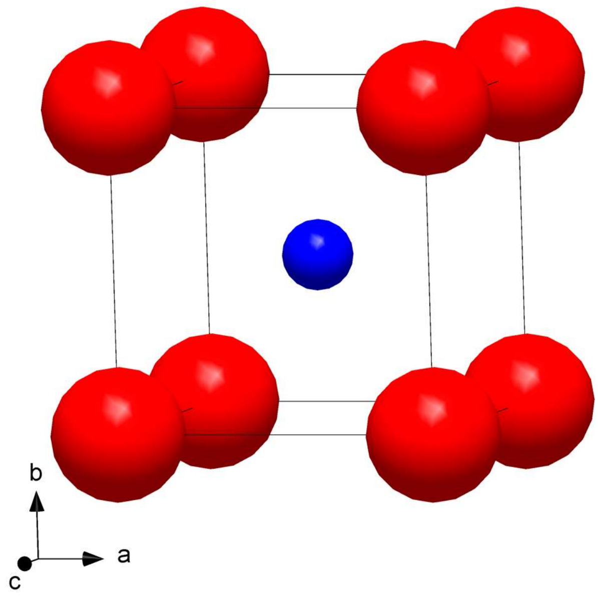


Supplementary Figure 5: Crystal structure of LaC at 150 GPa. The red and blue spheres represent lanthanum and carbon, respectively.

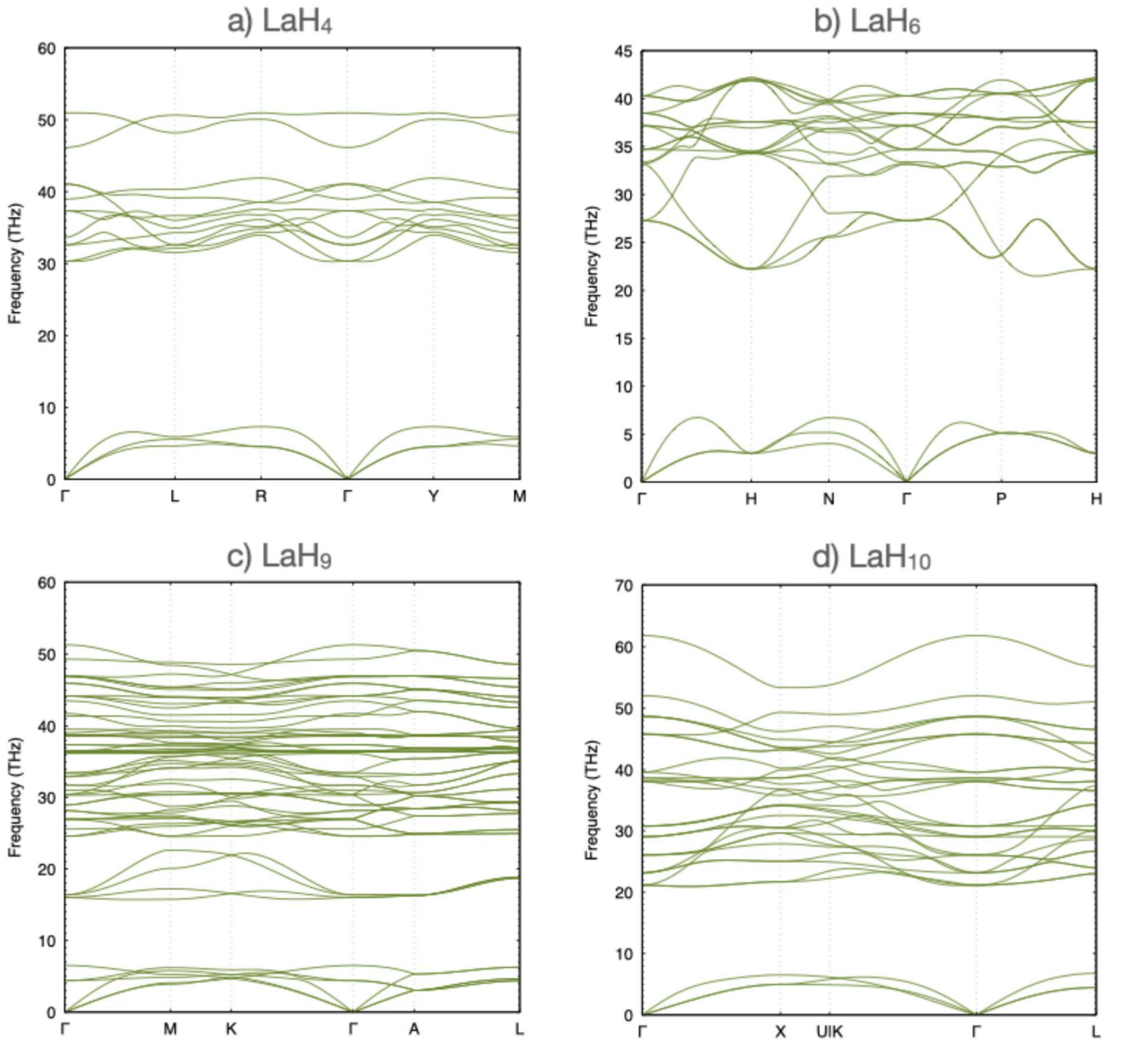


Supplementary Figure 6: Effective frequencies obtained from TDEP (anharmonic) sampling at 300 K for $LaH_4$, $LaH_6$, $LaH_9$, and $LaH_{10}$ for unit cell volumes corresponding to the experimental pressures of 150, 150, 140 and 140 GPa, respectively. All are positive definite, although they show imaginary frequencies at the purely harmonic level, with the exception of $LaH_4$ (see Supplementary Figure 9).

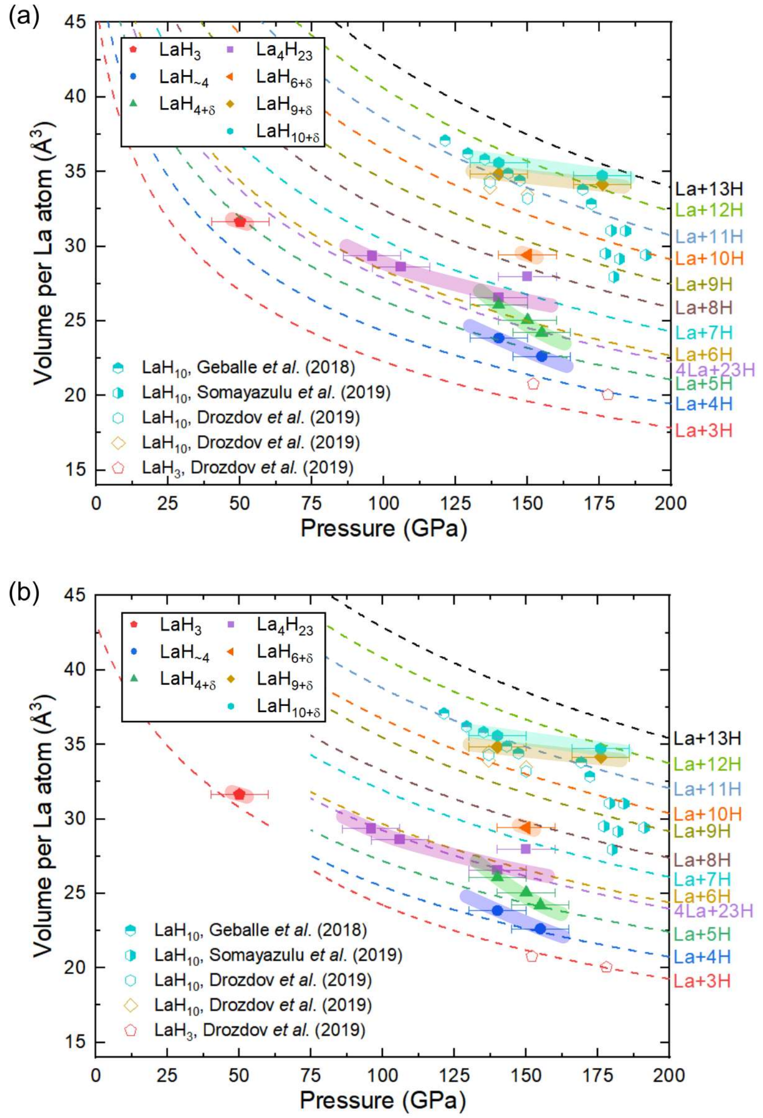


Supplementary Figure 7: Comparison of the experimental volume of the La-H compounds to that of hypothetical La-H compounds obtained through different approaches. (a) The unit cell volume per La atom as a function of pressure plotted for the seven synthesized lanthanum hydrides. The solid symbols represent the data obtained in this study. The error bars are the largest difference observed between the rhenium and the diamond pressure gauges (± 10 GPa). The colored dashed lines represent the pure element mixture, *i.e.* lanthanum plus three to thirteen hydrogen atoms, with the EoS of atomic hydrogen used[7]. Literature data for *Fm*-3*m* $LaH_{10}$ (empty and half-filled cyan hexagons) and *P*6$_3$/*mmc* $LaH_{10}$ (empty yellow diamond) and *Fm*-3*m* $LaH_3$ (empty red pentagons)[2,6,8]. (b) Same as Figure 2 (a) but with the colored lines now representing a pure element mixture using the volume per H atom derived from the DFT-calculated EoS of $LaH_3$, $LaH_4$, $La_4H_{23}$, $LaH_6$, $LaH_9$ and $LaH_{10}$. For $LaH_3$, the DFT relaxation shows a distortion of hydrogen atoms' position from the *Fm*-3*m* space group structure into a *R*-3*m* space group structure above 50 GPa. This *R*-3*m* space group structure has an EoS in agreement with the data points reported by Drozdov *et al.*[2] above 150 GPa.

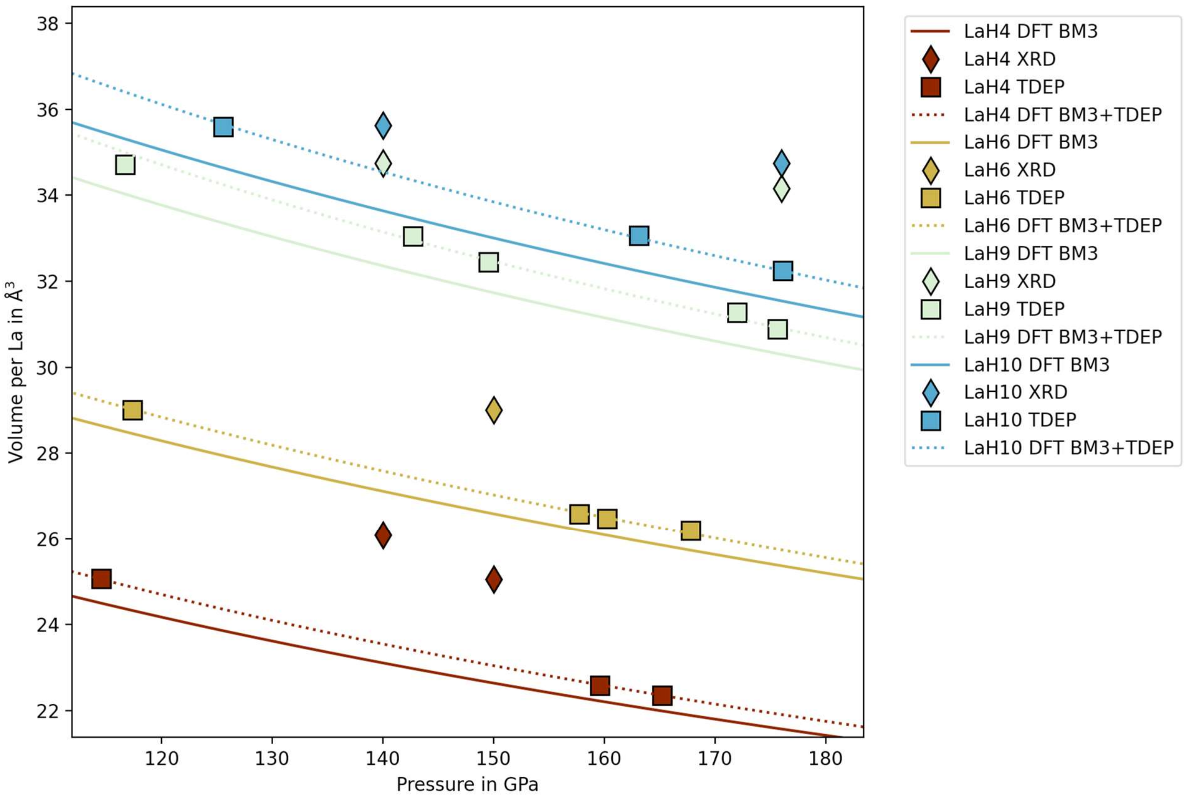


Supplementary Figure 8: Comparison of the pressure-volume dependency for $LaH_4$, $LaH_6$, $LaH_9$ and $LaH_{10}$. Diamonds are the XRD data presented in the tables above. Solid lines are third order Birch-Murnaghan EoS fits to the static DFT relaxations. Squares are finite temperature relaxations using TDEP at 300 K. Dotted lines are the static EoS fit shifted by the average pressure correction found in the finite temperature relaxations.

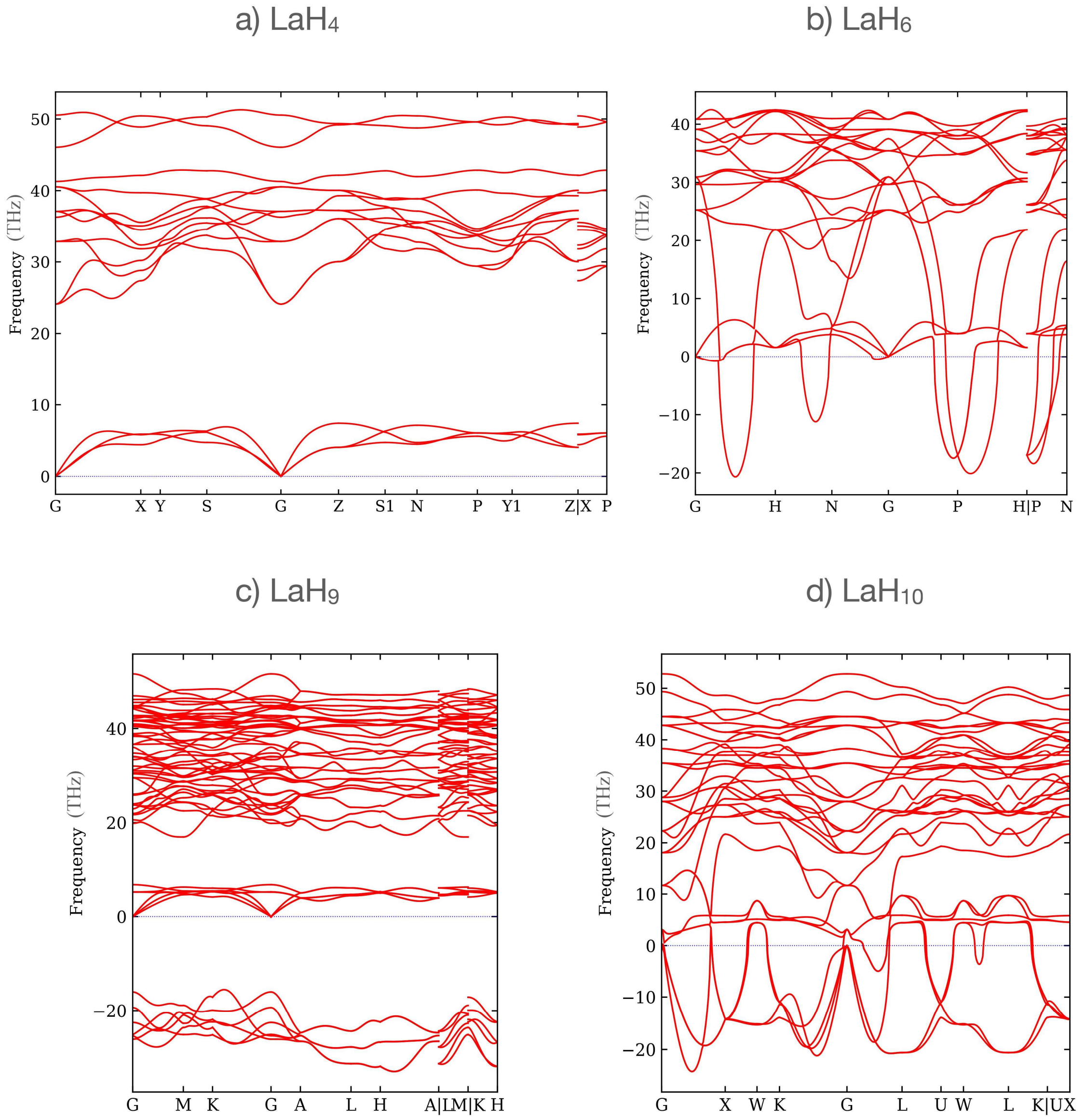


Supplementary Figure 9: Harmonic phonon dispersions for $LaH_4$, $LaH_6$, $LaH_9$, and $LaH_{10}$ obtained using Phonopy[9], for unit cell volumes corresponding to the experimental pressures of 150, 150, 140 and 140 GPa, respectively. The resulting phonon spectra show imaginary frequencies for all compounds with the exception of $LaH_4$.

## Supplementary References


1. Korst, W. L. & Warf, J. C. Rare Earth-Hydrogen Systems. I. Structural and Thermodynamic Properties. *Inorg. Chem.* **5**, 1719–1726 (1966).
2. Drozdov, A. P. *et al.* Superconductivity at 250 K in lanthanum hydride under high pressures. *Nature* **569**, 528–531 (2019).
3. Meier, T. *et al.* Nuclear spin coupling crossover in dense molecular hydrogen. *Nat. Commun.* **11**, 6334 (2020).
4. Ramachandran, G. K. *et al.* Synthesis and X-ray characterization of silicon clathrates. *J. Solid State Chem.* **145**, 716–730 (1999).
5. Semenok, D. V *et al.* Novel Strongly Correlated Europium Superhydrides. *J. Phys. Chem. Lett.* **12**, 32–40 (2021).
6. Geballe, Z. M. *et al.* Synthesis and Stability of Lanthanum Superhydrides. *Angew. Chemie Int. Ed.* **57**, 688–692 (2018).
7. Pépin, C. M., Geneste, G., Dewaele, A., Mezouar, M. & Loubeyre, P. Synthesis of FeH 5 : A layered structure with atomic hydrogen slabs. *Science (80-. ).* **357**, 382–385 (2017).
8. Chen, W. *et al.* Superconductivity and equation of state of lanthanum at megabar pressures. *Phys. Rev. B* **102**, 134510 (2020).
9. Togo, A. & Tanaka, I. First principles phonon calculations in materials science. *Scr. Mater.* **108**, 1–5 (2015).
